# To Beet or Not (Just) to Beet: Ecology, Virulence and Genomic Insights Into the Leaf Spot Disease Causative *Pseudomonas syringae* pv. *aptata*

**DOI:** 10.1111/mpp.70306

**Published:** 2026-06-30

**Authors:** Ivan Nikolić, Iva Rosić, Olja Medić, Tamara Ranković, Marina Sokić, Tanja Berić, Jelena Lozo, Slaviša Stanković

**Affiliations:** ^1^ University of Belgrade—Faculty of Biology Belgrade Serbia; ^2^ Center for Pathogen Biocontrol and Plant Growth Promotion University of Belgrade—Faculty of Biology Belgrade Serbia; ^3^ Institute of Physics Belgrade, National Institute of the Republic of Serbia, Laboratory for Experimental Astrobiology University of Belgrade Belgrade Serbia

## Abstract

**Taxonomy:**

Kingdom Bacteria; Phylum Proteobacteria; Class Gammaproteobacteria; Family Pseudomonadaceae; Genus *Pseudomonas*; Species *Pseudomonas syringae* species complex, Genomospecies 1, Phylogroup 02b.

**Biology:**

Gram‐negative, aerobic, motile, rod‐shaped with polar flagella, oxidase negative, arginine dihydrolase negative, levan production positive, potato rot negative, tobacco hypersensitivity positive.

**Virulence Factors:**

T3SEs, toxins (syringomycin, syringopeptin, syringolin, mangotoxin, tabtoxin).

**Disease Symptoms:**

*Pseudomonas syringae* causes disease symptoms on beet leaves characterised as circular or irregular, dark‐edged, white to light brown necrotic spots that merge and spread over the entire leaf surface. Young leaves become completely necrotic within 7 days after the first symptom appears.

**Host Range:**

Reported disease incidence mainly occurs on beets and cucurbits in temperate regions. Greenhouse trials showed an extremely wide host range for the strains isolated from diseased beets, causing disease on plants from nine different families and 16 different plant species.

**Disease Control:**

Preventive measures focus primarily on sanitary and cultural practices. Integrated control programmes benefit from the regular application of copper formulations. The susceptibility of sugar beet cultivars to leaf spot disease varies, but no cultivar is completely resistant to the pathogen. Biological control may involve beneficial bacteria that suppress pathogens via metabolites and resource competition or by inducing systemic resistance in beet plants.

**Useful Websites:**

https://www.pseudosonseed.org/chenopods‐diseases/; https://www.cabi.org/isc/datasheet/44993; https://www.ncbi.nlm.nih.gov/assembly/?term=pseudomonas%20syringae%20pv.%20aptata; https://www.uniprot.org/proteomes?query=pseudomonas%20syringae%20pv.%20aptata; https://pnwhandbooks.org/plantdisease/host‐disease/beet‐red‐beta‐vulgaris‐bacterial‐leaf‐spot.

## Graphical abstract

We profile *Pseudomonas syringae* pv. *aptata*, causing bacterial leaf spot on chenopods and cucurbits, highlighting epidemiology, virulence mechanisms and integrated disease management strategies.

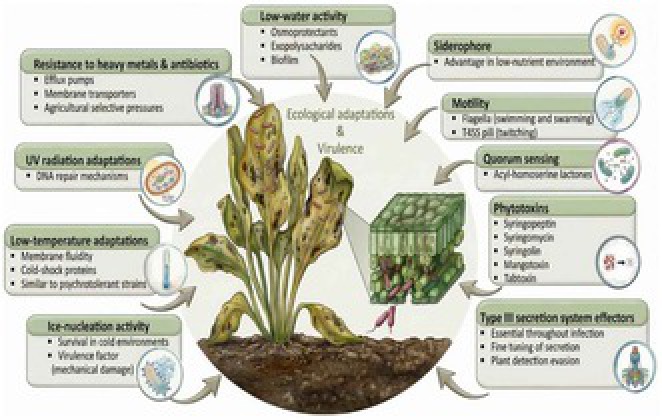

## 1 Introduction

*Beta vulgaris* (Chenopodiaceae) is a globally important crop species encompassing several major cultivated forms—sugar beet (primarily grown for sucrose production), Swiss chard and table beet/beetroot (cultivated as leaf and root vegetables) and mangel (used as a fodder crop) (Goldman and Navazio 2008). Sugar beet is the most widely grown and harvested among beet crops, with 281 Mt and 4.4 million ha harvested in 2024 (Nap et al. 2025), which explains why the majority of disease/epidemics reports come from sugar beet fields, although the same pathogens may infect other cultivated beet species as well. Fungal and bacterial diseases have long challenged beet cultivation, yet few pathogens illustrate the question of “to beet or not (just) to beet” as clearly as *Pseudomonas syringae* pv. *aptata*, the causal agent of bacterial leaf spot of beet. Although it belongs to the environmentally versatile and widely distributed *Pseudomonas syringae* species complex, this pathovar is traditionally associated with sugar beet. However, accumulating evidence indicates that *P. syringae* pv. *aptata* has a broader ecological and pathogenic role than previously recognised, infecting a range of non‐beet hosts and persisting outside beet plants. A holistic perspective on the foliar pathobiome (or pathocenosis) of beets clearly shows the dominance of fungal pathogens, led by *Cercospora beticola* (Esh and Taghian 2022). On the other hand, bacterial diseases in sugar beet have been documented for over a century, with *P. syringae* pv. *aptata* identified as the causal agent of leaf spot at the beginning of the 20th century (Brown and Jamieson 1913). Despite this long‐standing recognition, our current understanding of bacterial pathogens affecting sugar beet remains limited compared to that of fungal pathogens.

Recent observations, including frequent disease reports in the past two decades (Koike et al. 2003; Dutta et al. 2014; Stojšin et al. 2015; Arabiat et al. 2016; Rotondo et al. 2019; Nampijja et al. 2021; Popović Milovanović et al. 2026), suggest that *P. syringae* pv. *aptata* strains are re‐emerging as critical pathogens on beets, causing economically important outbreaks. *P. syringae* pv. *aptata* is a bacterial pathogen primarily associated with sugar beet ( *B. vulgaris* subsp. *vulgaris* var. *altissima*), where it causes leaf spot and blight symptoms that can lead to substantial yield losses under favourable environmental conditions. In addition to sugar beet, this pathovar has been reported to infect other cultivated forms of *B. vulgaris* , including table beet, beetroot and Swiss chard, indicating a broader host range within the species. Despite its long history of association with *B. vulgaris* crops, relatively little is known about the molecular basis of its pathogenicity and host specificity. In this review, we highlight recent genomic and molecular studies that are beginning to reveal how *P. syringae* pv. *aptata* interacts with its hosts, providing new opportunities to understand bacterial leaf spot disease in this important group of crops. Specifically, we profile *P. syringae* pathogenic on sugar beet and provide an overview of the latest research in order to elucidate the current status of *P. syringae* pv. *aptata* in the sugar beet pathobiome. This pathogen profile summarises current knowledge and advances in (i) distribution, taxonomy and phylogenomic status of *P. syringae* pv. *aptata* pathogenic on beets; (ii) epidemiology, cultivar susceptibility and host range; (iii) genomic and proteomic insights into pathogenic features of *P. syringae* isolated from sugar beet; (iv) genome features underlying ecological traits ensuring survival in the plant phyllosphere; (v) control management; and (vi) future research directions.

## 2 Distribution, Taxonomy and Phylogenomic Status

**FIGURE 1:**
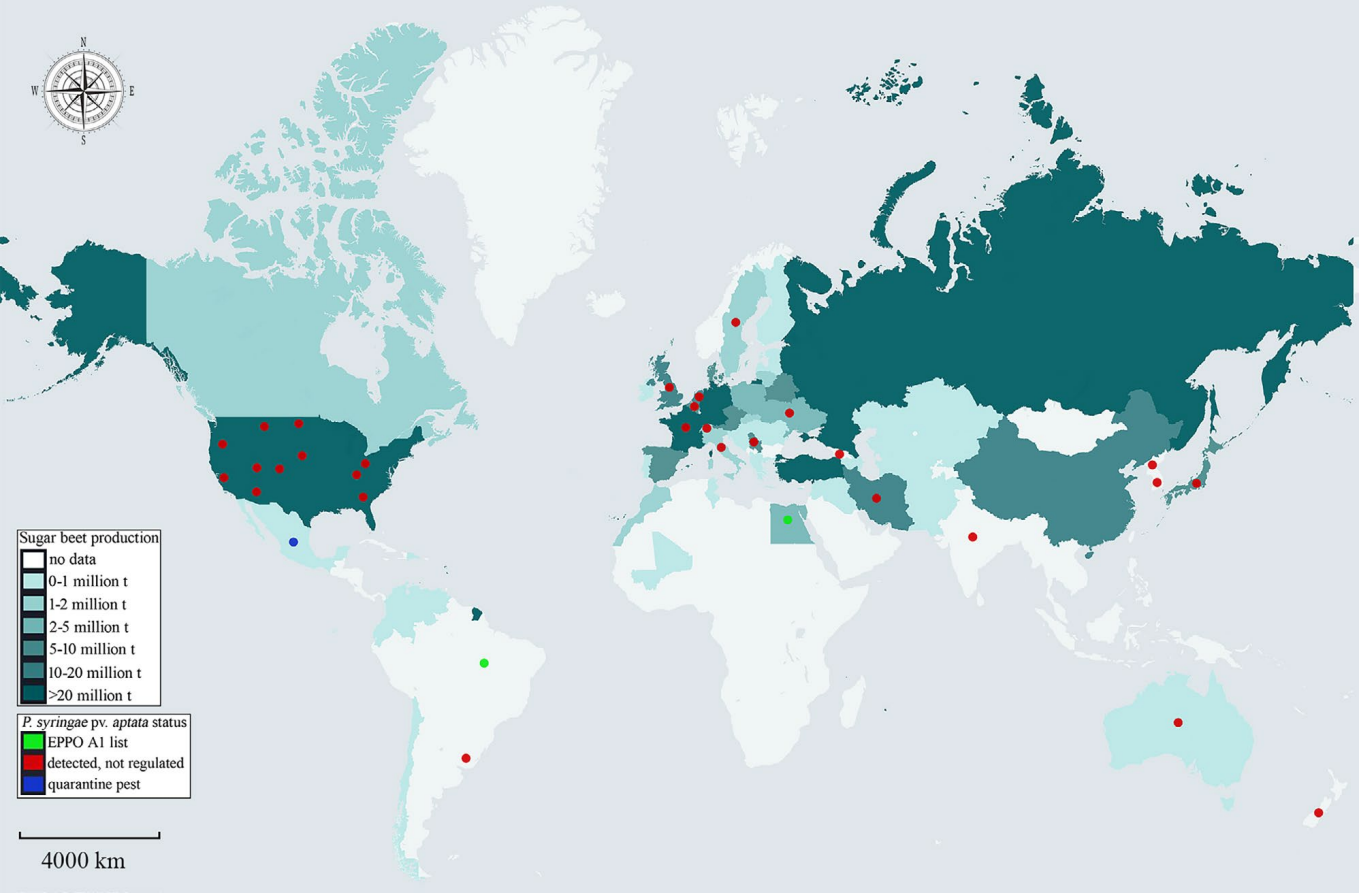
Worldwide co‐occurrence of reported *Pseudomonas syringae* pv. *aptata* outbreaks and sugar beet cultivation, emphasising spatial overlap between pathogen prevalence and agricultural intensity.

Originally, *P. syringae* pv. *aptata* was isolated as the causative agent of leaf spot disease on beet plants in North America, specifically in Utah and California, from sugar beet leaves (Brown and Jamieson 1913). As a causative agent of leaf spot disease on sugar beet, it has been detected in at least six continents (Figure 1): North America (United States: Arizona, California, Colorado, Georgia, Kentucky, Montana, North Dakota, Nebraska, Ohio, Oregon and Utah), South America (Uruguay), Europe (Belgium, France, Georgia, Italy, Netherlands, Russia, Serbia, Sweden, Switzerland, Ukraine, United Kingdom), Asia (Democratic People's Republic of Korea, India, Iran, Japan and South Korea) and Australia and New Zealand (Bradbury 1986; Morris et al. 2000; Koike et al. 2003; Nikolić et al. 2018; Sedighian et al. 2014). Additionally, *P. syringae* pv. *aptata* was added as an A1 pathogen on the EPPO list of pathogens in Egypt and Brazil, and was designated as a “quarantine pest” in Mexico in 2018 (https://gd.eppo.int/taxon/PSDMPT/categorization). Most reports come from countries with the largest sugar beet production (Figure 1), including the United States and EU countries, whereas information about *P. syringae* pv. *aptata* pathogenic on sugar beet from Russia, despite its status as one of the world's largest producers, remains scarce (Tarakanov et al. 2022).

Currently, *P. syringae* is referred to as a species complex, defined as a cluster of related monophyletic groups reflecting historical trends in bacterial classification, initially based on phenotypes and progressively on genotypes, including DNA–DNA hybridisation and phylogenetic analysis of housekeeping gene sequences (Berge et al. 2014). To date, approximately 64 pathovars have been identified that cause disease in a wide range of diverse plant species, although the majority are associated with crops. Among the many facets of species and strain diversity within the *P. syringae* complex, a large number of pathovars can be or should be revisited. This perspective suggests that modern revolution in molecular techniques, coupled with comprehensive pathogenicity assays, creates a strong necessity for revision of the pathovar concept within the *P. syringae* complex, especially through the lens of phylogroup 02 (PG02). Delineation of *P. syringae* strains pathogenic to sugar beet (mostly named as pv. *aptata*) has been investigated over the last two decades using techniques such as DNA–DNA hybridisation, housekeeping gene sequencing, molecular fingerprinting and, more recently, whole‐genome sequence comparisons (Gardan et al. 1999; Berge et al. 2014; Nikolić et al. 2018; Gomila et al. 2017; Baltrus et al. 2011; Ranković et al. 2023). In fact, strains isolated from leaf spot disease epidemics on sugar beet, together with the pathotype strain *P. syringae* pv. *aptata* CFBP1617, were grouped within PG02 (one of 13 phylogenetic groups within the *P. syringae* complex) based on multilocus sequence typing (MLST) analysis (Berge et al. 2014). Further subdivision within PG02 places the beet pathogenic strains in the 2b subgroup.

**FIGURE 2:**
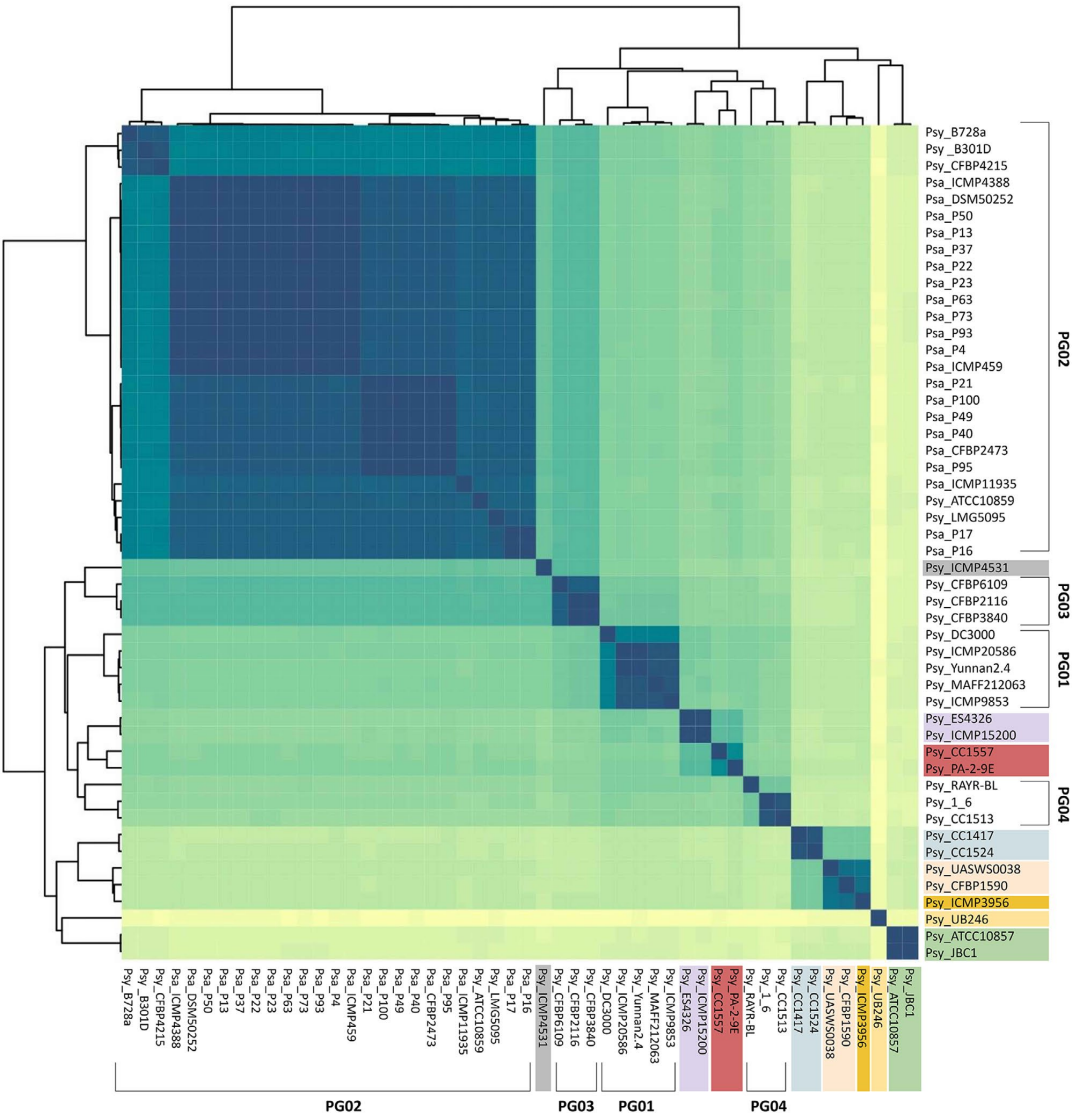
Comparative genomic analyses of all 21 publicly available genomes of *Pseudomonas syringae* pv. *aptata*, along with representative strains from each recognised *P. syringae* phylogroup, provide a clear picture of the phylogenetic placement of this pathovar. The ANI‐based phylogenetic reconstruction unequivocally places all pv. *aptata* isolates within PG02, revealing a striking genetic coherence among strains associated with sugar beet. Colour code: grey‐PG05, violet‐PG06, red‐PG07, light blue‐PG08, light orange‐PG09, dark yellow‐PG10, light yellow‐PG11, green‐PG13 (PG12 is not currently represented in publicly available genome databases).

Modern techniques, including whole genome sequencing and comparative genomics, enable more robust phylogenomic analysis within the *P. syringae* complex (Gomila et al. 2017) and confirm that strains pathogenic to beets belong to PG02 (Ranković et al. 2023). Beyond that, the constant expansion of genomic data resources has significantly improved our understanding of the diversity and evolutionary relationships within the *P. syringae* complex (Figure 2). Collectively, these findings reinforce the notion that PG02 represents the evolutionary and epidemiological nucleus of beet‐pathogenic *P. syringae* , underscoring its significance as a major lineage affecting sugar beet health and productivity.

## 3 Epidemiology and Host Range

**FIGURE 3:**
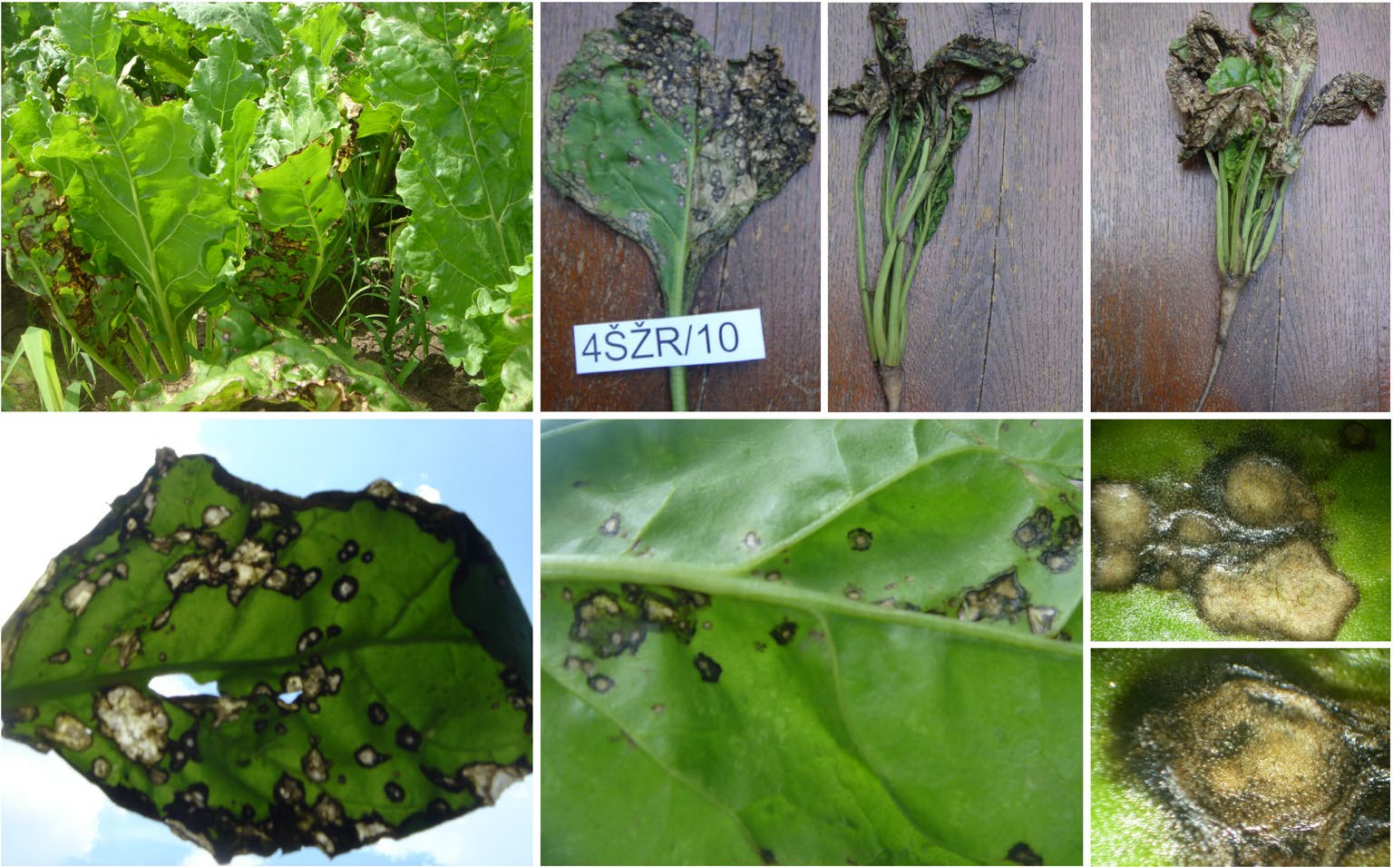
Bacterial leaf spot disease symptoms on sugar beet ( *Beta vulgaris* subsp. *vulgaris* var. *altissima*) in Serbian commercial fields at Vojvodina Province (Stojšin et al. 2015), associated with *Pseudomonas syringae* pv. *aptata* infection.

*Pseudomonas syringae* pv. *aptata* is the causal agent of bacterial leaf spot, a widespread disease that affects several agriculturally significant plant species. Among chenopods, its primary hosts are sugar beet ( *B. vulgaris* subsp. *vulgaris* convar. *vulgaris* var. *altissima*), Swiss chard ( *B. vulgaris* subsp. *cicla*), and beetroot ( *B. vulgaris* subsp. *vulgaris* var. *vulgaris*) (Koike et al. 2003; Sedighian et al. 2014; Stojšin et al. 2015). Infected plants typically exhibit irregularly shaped necrotic leaf spots, measuring 5–20 mm in diameter, characterised by dark, well‐defined borders, while the central areas range in colour from brown to grey (Figure 3). Such symptoms can easily be mistaken for those caused by the fungal pathogen *Cercospora beticola*, one of the most aggressive and harmful pathogens affecting sugar beet crops (Hallau et al. 2018). However, lesions caused by *C. beticola* lack the distinct dark, glassy margins characteristic of bacterial infections and generally appear later in the growing season, typically during July and August in the northern hemisphere (Weiland and Koch 2004; Rangel et al. 2020).

During the past decade, at least five disease outbreaks on beet plants caused by *P. syringae* pv. *aptata* have been reported worldwide (Dutta et al. 2014; Stojšin et al. 2015; Arabiat et al. 2016; Rotondo et al. 2019; Nampijja et al. 2021). The actual number of *P. syringae* pv. *aptata* epidemics may be substantially higher, as disease symptoms closely resemble those of fungal leaf spot disease, likely leading to misdiagnosis (Rangel et al. 2020). However, the key distinguishing factor is the timing of disease occurrence and environmental conditions favouring bacterial infection. So far, reported epidemics on beets caused by *P. syringae* pv. *aptata* have occurred in early spring (April–June), late autumn, or even during winter in greenhouse systems, consistently under comparable environmental conditions, such as low temperature and high humidity (Dutta et al. 2014; Stojšin et al. 2015; Arabiat et al. 2016). On the contrary, *Cercospora*‐mediated disease occurs primarily during summer under relatively high temperatures (Rangel et al. 2020). Thus, although the fungal pathogens dominate the beet pathobiome throughout the full vegetative period, *P. syringae* still finds a suitable window to establish itself within the beet pathobiome by exploiting favourable environmental factors. *P. syringae* pv. *aptata* epidemics are typically associated with cool, humid conditions that favour bacterial multiplication and dispersal through rain splash, overhead irrigation, or mechanical injury. Outbreaks have been documented in seedling nurseries, greenhouse production and field‐grown crops, often leading to substantial foliar damage, reduced photosynthetic area and diminished marketable yield in leafy types such as Swiss chard. For example, in the Salinas Valley of California, the pathogen was isolated from commercial Swiss chard fields over several growing seasons (1999–2003) and shown to be seed‐borne and disseminated via water‐splash (Koike et al. 2003). Subsequent, extensive genetic studies revealed distinct pathogen genotypes in beet/chard seed‐production regions of the US Pacific Northwest, highlighting the potential for asymptomatic colonisation in seed lots and later foliar outbreaks (Nampijja et al. 2021). In greenhouse evaluations conducted in Wisconsin, United States, involving 20 diverse table beet cultivars and breeding lines, spray inoculation of *P. syringae* pv. *aptata* revealed substantial genotypic variation in susceptibility to bacterial leaf spot. Furthermore, quantitative assessments showed that certain cultivars and plant introductions maintained consistently lower disease severity, reflecting partial resistance or tolerance, while others exhibited pronounced susceptibility (Gaulke and Goldman 2022). These responses remained stable across both growth stages and repeated experimental trials, suggesting that resistance to *P. syringae* pv. *aptata* is heritable and that selection for more resistant genotypes could represent a viable strategy for improving disease management in table beet. In general, disease incidence tends to be highest in regions with prolonged leaf wetness and moderate temperatures (15°C–25°C), under which the pathogen can survive epiphytically and initiate secondary infections. Although losses are generally less severe than in sugar beet, localised epidemics in table beet and chard production systems underscore the pathogen's broad host adaptability and the need for effective seed sanitation, resistant cultivars and cultural practices to limit disease spread. Together, these findings emphasise that while sugar beet is commonly implicated, the foliar crops and seed‐production systems of beet and chard are also at substantial risk, underscoring the need for vigilant seed testing, cultivar selection and host‐resistance strategies in these sectors.

Aside from chenopods, severe outbreaks caused by *P. syringae* *sensu stricto* were documented in France in 1993, where *P. syringae* pv. *aptata* caused up to 80%–100% yield loss in cantaloupe ( *Cucumis melo* var. *cantalupo*) (Morris et al. 2000). Plant‐originated isolates of *P. syringae* obtained from beets are heterogeneous, both phenotypically and genotypically, although they tend to form clonal populations in colonised areas (Morris et al. 2000). Symptoms observed in cucurbits include lesions on leaves, stems and fruits. Leaf spots are initially oily, dark green and small. Subsequently, affected tissues turn yellow and necrotic, eventually appearing brown to black. Stem lesions are initially wet to oily, later turning brown and crumbling slightly. Melon fruit symptoms appear as translucent, circular to slightly depressed spots, with a tendency to spread, while the central tissues gradually turn brown to completely black. Among cucurbits, greenhouse experiments confirmed pathogenicity on leaves of melon, zucchini, cucumber and watermelon (Morris et al. 2019). Besides chenopods and cucurbits, *P. syringae* pv. *aptata* has been reported to infect a range of monocot and dicot hosts, with disease documented on more than eight plant hosts, including pepper, parsley, onion, pea, cabbage, lettuce, tomato and spinach (Morris et al. 2019). Considerable variation in virulence has been observed, with strains showing low, intermediate, or most commonly, high virulence across diverse vegetable hosts (Morris et al. 2019).

In addition to its role as a crop pathogen, *P. syringae* pv. *aptata*, which is pathogenic to sugar beet and melon, has been found in both agricultural and natural environments (Riffaud and Morris 2002; Anteljević et al. 2025). Recent findings show that *P. syringae* isolates recovered from irrigation channels in the Danube River Basin exhibited 100% sequence identity in the partial citrate synthase (*cts*) gene to isolates of *P. syringae pv. aptata* obtained from infected sugar beet fields located close to these channels (Anteljević et al. 2025). This perfect match strongly supports the hypothesis that water‐borne populations of PG02 strains serve as reservoirs of plant‐pathogenic *P. syringae* pv. *aptata* and that PG02 plays a pivotal role in the regional epidemiology of this disease. Similar findings for PG02 populations were reported in the Durance River basin in southern France (Morris et al. 2023). These observations highlight the potential threat this pathogen poses not only to sugar beet but also to a broader range of susceptible plant hosts via multiple dissemination routes, including both seed‐borne and irrigation‐borne transmission. Consequently, continued surveillance and research into its epidemiology and virulence are essential for early detection and effective disease management.

The traditional *P. syringae* pathovar system, while invaluable for agricultural diagnostics, provides only limited insight into the biological and evolutionary relationships among strains. This limitation is particularly evident in the case of *P. syringae* pv. *aptata*, which exhibits a remarkably broad host range, infecting multiple beet subspecies (including sugar beet, table beet and Swiss chard) as well as several non‐beet hosts (Morris et al. 2000, 2019; Ramírez et al. 2023). Within the *P. syringae* species complex, pv. *aptata* strains are distributed within PG02, yet display substantial genomic and phenotypic diversity despite sharing the same pathovar designation (Berge et al. 2014; Gomila et al. 2017). This reflects a broader issue across the complex, where pathovar names often poorly correspond to phylogenetic structure: closely related strains may be assigned to different pathovars, while isolates of a single pathovar may be scattered across multiple phylogroups (Morris et al. 2019). Such incongruence complicates efforts to identify the genetic determinants of host specificity and virulence (Dillon et al. 2019). While some pv. *aptata* isolates appear highly host‐adapted, others act as host generalists, colonising a wide range of plant species under favourable conditions (Monteil et al. 2016; Ramírez et al. 2023). Moreover, the close genetic relatedness between crop‐associated and environmental isolates within PG02 suggests a continuum between epiphytic and pathogenic lifestyles, highlighting the dynamic nature of host–microbe–environment interactions in disease emergence (Monteil et al. 2016). The increasing availability of genomic data now provides an unprecedented opportunity to resolve these ambiguities. However, inconsistencies in genome annotation, incomplete metadata and outdated taxonomic assignments in public databases continue to obscure meaningful comparisons (Chorlton 2024). Accordingly, there is a pressing need for a comprehensive, genome‐based re‐evaluation of pathovar boundaries within PG02, particularly for *P. syringae* pv. *aptata*, to establish a more biologically coherent framework for understanding host range evolution and pathogenic diversity within this lineage (Dillon et al. 2019; Mulet et al. 2024).

## 4 Genomic Insights into the Survival and Virulence Strategy

### 4.1 Survival

**FIGURE 4:**
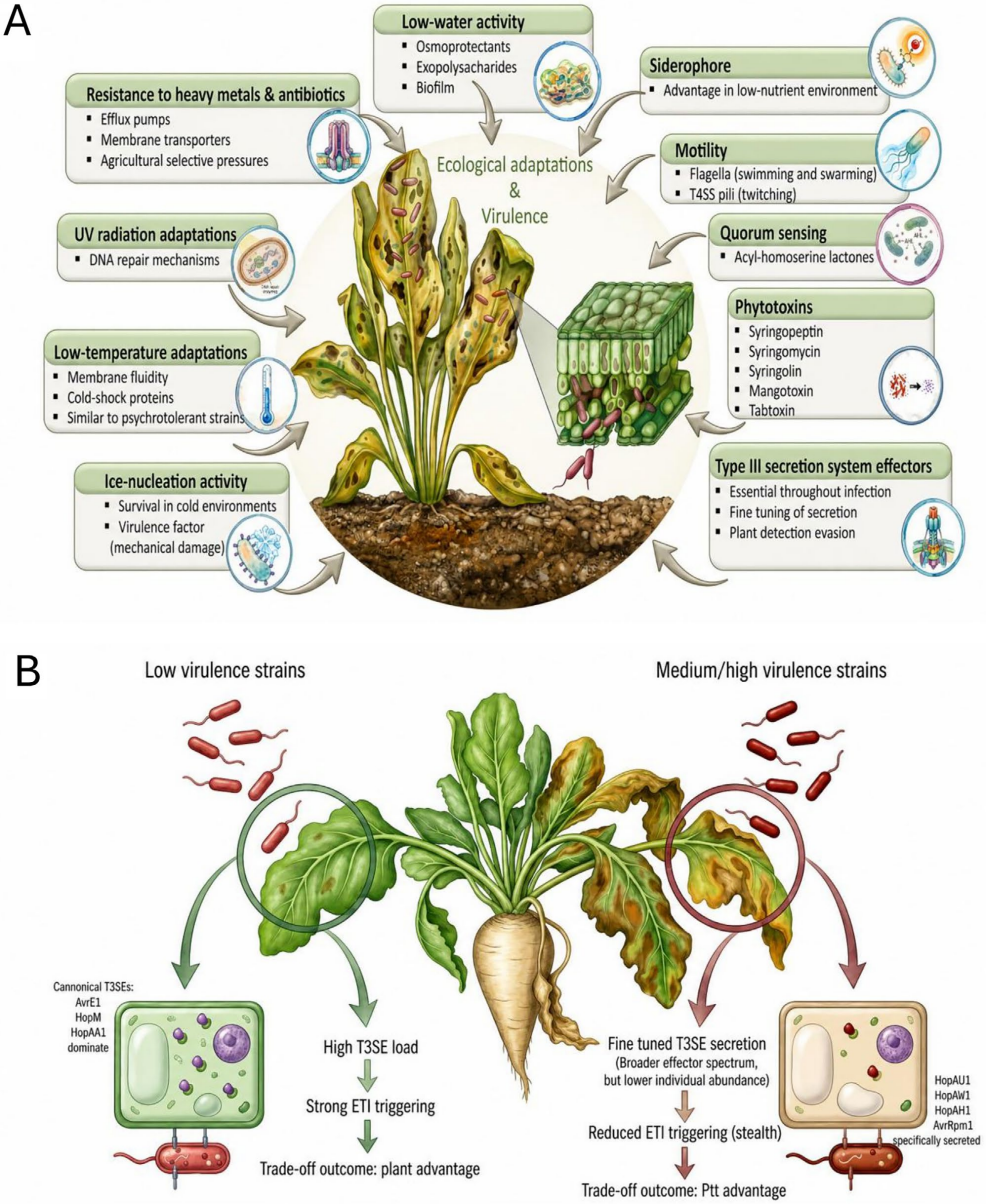
Virulence strategy of *Pseudomonas syringae* pv. *aptata*. (A) Key ecological adaptations that support environmental persistence, host colonisation and suppression of plant defences. (B) Differences in the type III secretion system (T3SS), associated effector repertoires and strategies that distinguish low‐, medium‐ and high‐virulence strains, and their contributions to disease severity.

The transition from a benign or commensal member of the phyllosphere to a pathogenic threat is driven by environmental factors, genetic diversification within the *P. syringae* complex, and host–pathogen interactions specific to sugar beet. Survival in the phyllosphere requires robust adaptation to UV radiation, fluctuating temperatures and osmotic stress caused by limited water availability (Figure 4A).

The effect of solar UV radiation has been intensively studied in phyllosphere ecology, with adaptation primarily linked to the ability of bacterial cells to repair DNA damage (Jacobs et al. 2005; Thapa and Prasanna 2018). Genome sequences of *P. syringae* pv. *aptata* strains revealed multiple genes involved in photoreactivation (*phrB*), mismatch repair (*mutSL*), the SOS response (*umuDC*, *yedK*), nucleotide excision repair (*uvrABC*) and double‐strand break repair (*recA*, *recFON*). Although these pathways are not specific to *P. syringae* or phyllosphere‐associated bacteria and are widely distributed among diverse bacterial taxa, their presence likely contributes to the ability of *P. syringae* to withstand UV stress and persist in the highly exposed phyllosphere environment. Additionally, low‐temperature adaptation further supports ecological fitness, suggesting that *P. syringae* pv. *aptata* strains can survive at low temperatures despite seasonal or daily fluctuations. Draft genome sequences revealed coding sequences associated with cold adaptation, such as genes for cold shock proteins (*capB*, *cspD*), ice nucleation protein (*inaZ*), aspartate aminotransferase (*aspC*), or genes for cold‐associated RNA proteins (*mnmE*) also found in the psychrotolerant *Pseudomonas* sp. strain Lz4W (Janiyani and Ray 2002; Sundareswaran et al. 2010; Pavankumar et al. 2018). Ice nucleation activity (INA) serves a dual role: it acts as a survival mechanism in cold environments and as a virulence factor that induces frost injury, facilitating bacterial entry into the apoplast (de Araujo et al. 2019). This may be particularly relevant in temperate regions, where disease occurrences are reported mainly in spring or winter under unstable temperature conditions. In contrast, the dominant fungal leaf spot pathogen of sugar beet, *C. beticola* , proliferates under warm and humid summer conditions (90% humidity, day 27°C–32°C, night > 17°C) (Rangel et al. 2020). Cold adaptation may therefore allow *P. syringae* pv. *aptata* to occupy ecological niches less favourable to competing pathogens, contributing to its prevalence within the beet pathobiome.

Adaptation to osmotic stress involves the accumulation of compatible solutes through biosynthesis and uptake (Brauer et al. 2023). Genomic analyses revealed genes associated with alginate production (*alg8ABDEE2GJKLX*), glycine/betaine/proline/choline transport systems (*proPSVY*, *betAT2*, *opuAA*–*AB*, *ousXY*), trehalose biosynthesis (*treSY*) and osmoprotective proteins (*osmEVWXY*). Alginate, a characteristic exopolymer of *P. syringae* , enhances bacterial aggregation and contributes to desiccation tolerance (Freeman et al. 2013). Transport systems enabling uptake of compatible solutes, such as choline, glycine and betaine, from plant tissues, soils, sediments and aquatic environments further expand ecological versatility. These osmoadaptive traits likely support persistence in agroecosystems, including irrigation systems and environmental reservoirs, where pv. *aptata* has been detected. Resistance to heavy metals and antibiotics further enhances ecological plasticity. Copper resistance genes (*copABCD*) and multicopper oxidase *mmcO* were detected in pv. *aptata* genomes, together with genes conferring resistance to arsenic and other metals. Multiple multidrug resistance transporters and efflux systems were also identified, including *tetA* and *tetC* tetracycline resistance genes and determinants conferring resistance to penicillin, chloramphenicol, bicyclomycin, polymyxin and bleomycin. The presence of these genes suggests exposure to agricultural selective pressures and may contribute to persistence in soils, irrigation systems and treated fields. Such resistance determinants increase ecological fitness and may promote the formation of stable environmental reservoirs.

Together, these ecological survival traits form the foundation for persistence within the phyllosphere and environmental reservoirs. However, persistence alone does not result in disease. The transition from an environmentally resilient epiphyte to an active pathogen requires coordinated activation of virulence determinants that enable host entry, immune suppression and apoplastic colonisation.

### 4.2 Virulence

Members of the *P. syringae* species complex possess a number of virulence factors involved in various stages of disease development (Pontes et al. 2020). In pv. *aptata*, the transition from epiphytic persistence to endophytic colonisation is mediated by tightly regulated motility, secondary metabolite production and secretion systems. When *P. syringae* pv. *aptata* shifts its life cycle from epiphytic to endophytic, the most prominent virulence factor, the type III secretion system (T3SS), is used for a plethora of roles in pathogenic manners, similar to other members of the *P. syringae* complex (O'Brien et al. 2011). The diversity and function of T3SS effector proteins in *P. syringae* reach enormous proportions compared with other gram‐negative bacteria, including both animal and plant pathogens. Further information about the diversity and evolution of the effectorome of the whole *P. syringae* species complex can be found in Bundalovic‐Torma et al. (2022).

A holistic view of the T3SS in *P. syringae* pv. *aptata* reveals a finely balanced interplay between the genomic repertoire and regulated effector secretion that underpins its pathogenic success on sugar beet. From genomic perspective, all pv. *aptata* strains harbour the core *hrp* (hypersensitive reaction and pathogenicity) pathogenicity island, which encodes the structural and regulatory machinery of the T3SS as well as a conserved “core” effector set (e.g., AvrE, HopI, HopM1, HopAA) (Table 1).

**TABLE 1:** Comparative genomic overview of virulence‐associated genes encoding type III secretion system effectors (T3SEs) in *Pseudomonas syringae* pv. *aptata* strains pathogenic on sugar beet.

Strain	T3SE																																							
	*AvrE*	*AvrRpm1*	*HopAA1	*HopAG1*	*HopAH1	*HopAH2*	*HopAI1*	*HopAU1a*	*HopAZ1*	*HopBA1	*HopBF1	*HopC1	*HopH1*	*HopI1*	*HopJ	*HopJ1*	*HopM1	*HopO1‐1*	*HopPmaK	*HopT1‐1*	*HopW*	*HopX+*	*HopZ3*	*HopAK1	*HrpK	*HopAC1*	*HopAE1*	*HopA1*	*HopAW1*	*HrpA	*HrpW	*HrpW1*	*HopAV1*	*AvrPphvF*	*HopAT1*	*AvrB3*	*XopAD*	*AvrXv3*	*AvrRps4*	*HopBC1
P4	+	+	+				+			+	+				+								+																	
P13	+		+				+			+					+								+																	
P16	+								+	+	+			+									+	+		+				+										
P17	+		+		+				+	+	+	+		+	+		+		+		+		+	+	+					+	+									+
P21	+	+	+					+			+				+						+			+	+	+	+		+	+	+		+							
P22	+		+				+			+	+				+								+																	
P23	+		+				+				+				+								+																	
P37	+		+				+			+	+				+								+																	
P40	+		+								+										+																			
P49	+		+					+			+				+						+																			
P50	+		+				+	+		+	+				+								+																	
P63	+		+				+			+	+				+								+																	
P73	+		+				+			+	+				+								+																	
P93	+		+				+			+	+				+								+																	
P95	+		+								+				+						+																			
P100	+		+					+							+						+																			
CFBP2473	+		+					+							+						+																			
DSM50252	+		+				+				+				+								+	+	+	+														
ICMP11935	+		+			+	+							+		+	+	+	+	+		+		+	+	+	+	+			+	+	+	+	+	+	+	+	+	
ICMP4388	+		+	+	+	+			+	+		+	+	+			+							+		+						+								
ICMP459	+		+	+	+				+	+	+	+	+	+		+							+	+	+															

*Note:* Publicly available genome sequences were analysed; asterisks indicate experimentally validated secretion of specific T3SEs. Colour shading corresponds to effectors predicted using the EffectorPredictor tool and documented in Ranković et al. (2023).

Yet beyond this conserved backbone, strain‐level variations emerge: different *P. syringae* pv. *aptata* isolates carry approximately 16 to 25 open reading frames (ORFs) encoding additional putative T3SS effectors, indicating flexible expansion and contraction of effector repertoires across strains (Ranković et al. 2023). From a secretome and functional secretion perspective under apoplast‐mimicking conditions, secretion of 23 distinct T3SS effectors has been demonstrated in at least some pv. *aptata* strains, confirming that most genomic candidates are functional at the protein secretion level (Nikolić et al. 2023). Interestingly, the low‐virulence strains paradoxically secreted the majority of canonical effectors at high abundance, whereas medium/high virulence isolates secreted a broader but individually lower abundance effector spectrum. Among these strain‐specific secreted effectors, a suite of four (HopAU1, HopAW1, HopAH1, AvrRpm1) was secreted only by the medium/high virulence group, suggesting a role in more aggressive phenotypes (Table 1). This dual view, genomic potential plus secretion profiling, supports a model in which *P. syringae* pv. *aptata* does not merely rely on maximal effector load, but instead must finely tune which effectors are secreted, and in what quantity, to evade the host's effector‐triggered immunity (ETI). These observations suggest that overabundant secretion of ETI‐eliciting effectors may reduce virulence, whereas more restrained and selective secretion may enhance virulence through stealth (Figure 4B). Together, the genomic complement of effector genes and the secretion dynamics of T3SS effectors constitute a stratified and adaptive virulence strategy in *P. syringae* pv. *aptata* on sugar beet, bridging genotype with phenotype in host–pathogen interactions. Comparable patterns have been observed in other plant‐pathogenic bacteria. Experimental evolution studies in *P. syringae* pv. *actinidiae* indicate that effector repertoires function as integrated systems in which host and context‐dependent interactions determine competitive success and favour retention of the full repertoire (Hemara et al. 2025). Similarly, in *Ralstonia solanacearum* , virulence and host range are shaped by the coordinated activity of multiple T3SS effectors rather than single dominant determinants (Landry et al. 2020; Cong et al. 2023), and in *Xanthomonas* spp., effector composition correlates strongly with host specificity and pathogenic fitness (Chen et al. 2024). The specialisation of *P. syringae* pv. *aptata* to sugar beet appears to reflect a unique adaptation of its T3SS effector repertoire compared to other *P. syringae* pathovars infecting diverse hosts. While core T3SS components and effectors are widely conserved across *P. syringae* , host specificity often arises from differences in effector content and secretion regulation. For instance, pathovars such as *P. syringae* pv. *tomato* and pv. *phaseolicola* typically deploy effectors like HopQ1 and AvrPtoB (Li et al. 2013; Liu et al. 2022), which are absent or unexpressed in *P. syringae* pv. *aptata*. Conversely, *P. syringae* pv. *aptata* secretes effectors such as HopAU1 and HopAW1 that are rarely found or functionally silent in other pathovars. This divergence may reflect adaptation associated with evasion of sugar beet immune surveillance mechanisms. Notably, sugar beet T3SS‐triggered immunity may recognise certain highly conserved effectors, driving *P. syringae* pv. *aptata* strains to adopt a more variable and regulated secretion profile. Comparative analyses underscore that high‐virulence *P. syringae* pv. *aptata* strains secrete a broader but lower‐abundance effector mix, a strategy that likely minimises detection while preserving suppressive function. In contrast, pathovars targeting solanaceous or leguminous hosts may rely on fewer, high‐potency effectors that are finely tuned to those plant immune systems (Lindeberg et al. 2009). This suggests that host adaptation in *P. syringae* is not only a matter of effector presence or absence but of secretion dynamics, abundance thresholds and interaction with host‐specific resistance genes. Prior to this, an early genomic record of *P. syringae* pv. *aptata* DSM50252 (Baltrus et al. 2011) had been annotated as carrying only about 12 T3SS effector ORFs, a comparatively modest repertoire among *P. syringae* pathovars. That low‐catalogue estimate probably under‐represented the true effector potential, partly due to limitations in prediction methods or incomplete sampling of accessory genes. Over time, bioinformatic predictions improved, and machine‐learning pipelines (e.g., Effectidor) applied across *P. syringae* genomes (including pv. *aptata*) increased the predicted repertoire (Wagner et al. 2022). Despite these genomic advances, functional validation of T3SS in pv. *aptata* remained limited. Earlier studies focused mainly on presence/absence and comparative inference, without direct assays of effector secretion, host translocation, or transcriptional regulation *in planta* for *P. syringae* pv. *aptata*. Nevertheless, genome comparisons laid a foundation: variation in effector content, especially accessory genes beyond the canonical hrp/Hrp‐associated core (e.g., *avrE*, *hopM*, *hopAA*), probably shapes strain‐level virulence differences. The discovery of AvrRpm1, HopAW1 and HopAU1 in *P. syringae* pv. *aptata* P21 (Table 1) is particularly intriguing because these effectors are more commonly seen in other *P. syringae* pathovars (McAtee et al. 2018; Fujikawa and Sawada 2019; Hemara et al. 2024).

In addition to T3SS‐mediated effector delivery, virulence in *P. syringae* pv. *aptata* is reinforced by complementary mechanisms, including flagella‐ and type IV pili‐dependent motility (Ichinose et al. 2016), biofilm formation through extracellular polysaccharides such as alginate and levan (Shao et al. 2021), siderophore‐mediated iron acquisition (Huang et al. 2022), quorum sensing via acyl‐homoserine lactone signalling (Yang et al. 2023) and phytotoxin production.

Phytotoxins in *P. syringae* are generally considered non‐host specific virulence factors, with most phylogroups encoding one or a limited number of toxins, whereas representatives of PG02 can harbour genes for the production of up to five different phytotoxins per strain (Dillon et al. 2019). PG02 strains are therefore characterised by a particularly diverse arsenal of these virulence factors with distinct strategies, including proteasome inhibition (syringolin), hormone mimicry (auxin, coronatine), interference with host metabolic processes (tabtoxin, mangotoxin, phaseolotoxin) and membrane disruption by pore formation (lipopeptide toxins syringomycin and syringopeptin) (Dillon et al. 2019; Grenz et al. 2025). Within pv. *aptata*, genes for syringomycin and syringopeptin represent the most consistently detected phytotoxin‐associated genes (Figure 4A), in agreement with their broad distribution across clade 2b and their frequent co‐occurrence in analysed strains (Baltrus et al. 2011; Dillon et al. 2019; Ranković et al. 2023). Additionally, syringomycin and syringopeptin are key virulence determinants that are strongly associated with broad host pathogenicity (Grenz et al. 2025). In contrast, syringolin biosynthetic genes are less common in clade 2b (Zeng et al. 2026), although they have been confirmed in pv. *aptata* (Baltrus et al. 2011). In addition, the mangotoxin biosynthetic operon (*mbo*) has been identified in several pv. *aptata* type strains (Carrión et al. 2013).

Additionally, *P. syringae* pv. *aptata* has been shown to exhibit type VI secretion system (T6SS) activity under apoplast‐like conditions (Nikolić et al. 2023), suggesting a potential role in virulence, consistent with findings reported for other *P. syringae* pathovars (Sarris et al. 2010; Wang et al. 2021). These factors contribute to successful surface colonisation, apoplast modification, suppression of plant immunity and nutrient acquisition. Their expression is tightly regulated by complex transcriptional and regulatory networks, ensuring that virulence determinants are deployed in coordination with environmental conditions and host‐derived signals (Xin et al. 2018; McAtee et al. 2018).

## 5 Control Management and Cultivar Susceptibility

Plant diseases caused by bacteria are frequently under‐recognised compared to those caused by fungi, insects, or nematodes (Mansfield et al. 2012; Rai and Rai 2025). This is particularly relevant for *P. syringae* pv. *aptata*, whose leaf spot symptoms on sugar beet closely resemble those caused by *C. beticola* , as discussed earlier in this manuscript. Although experienced agronomists, supported by molecular and microbiological diagnostics, can distinguish between these pathogens, such diagnostic confirmation is not consistently performed in routine agricultural practice. Consequently, disease management responses are often delayed or nonspecific. Fungicides targeting *Cercospora* may be applied inappropriately, or bactericides may be used without a confirmed diagnosis, leading to inconsistent selection pressures on *P. syringae* pv. *aptata* populations and potentially accelerating the emergence of resistance (Sundin and Wang 2018). Therefore, management of *P. syringae* pv. *aptata* cannot rely solely on reactive chemical control.

Management of bacterial leaf spot disease in sugar beet relies on an integrated approach that combines sanitary and cultural practices, chemical applications and biological control, with particular emphasis on disease prevention by inhibiting *P. syringae* during its epiphytic life cycle stage. According to the plant disease management hexagon concept, protection, resistance, therapy, avoidance, exclusion and eradication, effective disease control requires consideration of the dynamic interplay among the pathogen, host plant, beneficial microbiota and environmental conditions (Yang et al. 2023). Exclusion and avoidance, therefore, represent logical entry points for sustainable management strategies.

Within this preventive framework, understanding sources of primary inoculum is particularly important. However, compared with other well‐characterised seed‐borne bacterial pathogens, seed transmission within the *P. syringae* species complex has received limited attention and sensitive detection methods remain a significant bottleneck (Goudarzi and Mortazavi 2020; Yang et al. 2023). As a result, low‐level contamination may remain undetected until environmental conditions such as cool temperatures, high humidity and prolonged leaf wetness favour rapid population expansion and epidemic development (Xin et al. 2018). Beyond seed‐borne inoculum, *P. syringae* pv. *aptata* persists in plant debris, weeds, irrigation water and as epiphytic populations on asymptomatic hosts (Morris et al. 2013), creating a complex and often overlooked network of environmental reservoirs. Because epiphytic populations on the phylloplane constitute the immediate source of infection, strategies aimed at limiting bacterial multiplication on leaf surfaces are particularly important for effective disease suppression.

Although no sugar beet cultivars are completely resistant to *P. syringae* pv. *aptata*, studies have reported variability in susceptibility. Four commercial sugar beet cultivars commonly grown in Serbia due to their resistance to *Cercospora* and *Rhizoctonia* showed variability in leaf spot disease symptom severity after infection with *P. syringae* pv. *aptata* (Nikolić et al. 2018). Additionally, differences in susceptibility to *P. syringae* pv. *aptata* among table beet ( *B. vulgaris* subsp. *vulgaris*) and Swiss chard ( *B. vulgaris* subsp. *cicla*) cultivars, breeding lines and plant introductions have also been reported under greenhouse conditions (Gaulke and Goldman 2022; Sharma et al. 2024). The development of *B. vulgaris* cultivars resistant to *P. syringae* pv. *aptata* remains an ongoing process, as the genetic basis of resistance is gradually being elucidated with recent identification of quantitative trait loci (QTLs) on chromosome 1 (Chr1_61344476) associated with disease response (Morrison and Goldman 2025). Identification of loci associated with resistance to disease caused by *P. syringae* pv. *aptata* demonstrates that promising genomic targets are emerging to guide breeding for resistant cultivars.

Currently, chemical treatments based on copper and antibiotics (streptomycin, oxytetracycline, gentamycin and oxolinic acid) are used to reduce phytopathogen populations on plant surfaces (Córdova et al. 2023). However, the emergence of both copper‐ and streptomycin‐resistant phytopathogenic strains has been continuously increasing (Vanneste et al. 2008; Aprile et al. 2021; Horuz et al. 2025). Copper‐based bactericides remain one of the few chemical options for managing bacterial leaf spot caused by *P. syringae* pv. *aptata* in beets. Various copper formulations (Group M1), including products based on copper hydroxide, copper oxychloride, copper sulphate, or cuprous oxide, are registered for use in beet production in the United States (Nampijja et al. 2025). However, copper treatments provide only limited control and must be applied preventively, as they are unable to eradicate the *P. syringae* pv. *aptata* once disease symptoms have developed. Copper‐based products remain the standard practice for disease management due to their broad‐spectrum bactericidal effect, but resistant *P. syringae* strains have been reported for 200 μg/mL and higher (Thomidis et al. 2025). In *P. syringae* pv. *syringae*, copper hyper‐resistance has emerged due to a large Tn*7*‐like transposon of chromosomal location, which harbours putative copper and arsenic resistance genes (Aprile et al. 2021), also found in pv. *aptata* strains. Besides the development of resistance in phytopathogenic bacteria, copper accumulation as a heavy metal poses a significant threat to plant health, non‐target organisms, soil quality and food safety (Córdova et al. 2023), which at some point also limits disease management caused by *P. syringae* pv. *aptata*.

Given the global trend toward reducing chemical pesticide use, biological control has emerged as an attractive and increasingly necessary alternative (Lahlali et al. 2022). Identifying biocontrol agents with a comparable ecological advantage is inherently difficult because effective suppression requires that the introduced organism persists, competes and survives long enough to express its antagonistic activity (Legein et al. 2020). The application of beneficial *Bacillus* spp. strains has so far proven to be a promising strategy for the management of *P. syringae* pv. *aptata*. Crude ethyl‐acetate extracts of lipopeptides from *Bacillus amyloliquefaciens* (SS‐12.6 and SS‐38.4) and *Bacillus pumilus* SS‐10.7 suppressed the co‐inoculated *P. syringae* pv. *aptata* P53 and reduced sugar beet leaf necrosis, while pure cultures of *B. amyloliquefaciens* SS‐12.6 achieved 60%–92% disease suppression (Nikolić et al. 2019). A subsequent study demonstrated that the soil‐dwelling *Bacillus velezensis* SS‐38.4 strain (previously *B. amyloliquefaciens* ) efficiently colonises the phyllosphere and provides significant biocontrol efficacy when sprayed before (preventive treatment) or after (curative treatment) the phytopathogen *P. syringae* pv. *aptata* P21 (Rosić et al. 2025). Population monitoring of the biocontrol strain and the phytopathogen, using strain‐specific molecular SCAR_38.4_ markers with digital PCR for absolute quantification and traditional colony counts, and correlating these data with biocontrol efficacy highlights the critical importance of preventive control measures during the phytopathogen's epiphytic life cycle stage. Suppression by SS‐38.4 appears to rely less on direct antibiosis and more on early niche occupation and extensive colonisation, particularly when applied before pathogen arrival (Rosić et al. 2025). This gives the biocontrol strain time to adapt to the phyllosphere environment and to establish stable populations on the leaf surface. Early establishment likely limits available space and resources for *P. syringae* pv. *aptata* and enables accumulation of antimicrobial compounds, collectively reducing pathogen expansion and subsequent symptom development. Similar findings have been reported in field experiments conducted in sugar beet, where indigenous *B. velezensis* strains demonstrated comparable effects (Milosavljević et al. 2026). In addition to *Bacillus* spp., sugar beet associated *Pseudomonas marginalis* OL141 and Orh26 strains were shown to induce resistance against *P. syringae* pv. *aptata*, with OL141 also promoting leaf growth, whereas these beneficial effects were absent in mutant strains with inactivated T3SS (Nedeljković et al. 2025a, 2025b). These emerging studies reveal an additional role of T3SS in nonpathogenic bacteria, extending beyond its well‐established function in virulence. Elucidation of T3SS‐dependent plant–microbe interactions may therefore offer novel opportunities for developing efficient disease management strategies based on a previously unknown biocontrol mechanism.

Effective management of bacterial leaf spot on sugar beet relies on integrated strategies. Beneficial *Bacillus* spp. strains, beneficial T3SS‐harbouring bacteria and bacteriophages offer promising biocontrol options, particularly when applied preventively, emphasising the potential for targeted and sustainable disease management. Effective protection against *P. syringae* pv. *aptata* ultimately requires a shift from reactive, symptom‐driven pesticide use to proactive ecological management, integrating prevention, biological control and carefully optimised chemical support within a sustainable framework (Lechenet et al. 2017).

## 6 Future Prospects

The “to beet or not (just) to beet” dilemma suggests that *P. syringae* pv. *aptata* should not be viewed merely as a beet‐associated pathogen, but as a part of a broader, environmentally persistent PG02 lineage whose virulence expression is context‐dependent rather than fixed. The key question is therefore not whether these bacteria can infect multiple hosts; they clearly can, but which ecological and molecular determinants govern the transition from environmental persistence to disease expression within specific agroecosystems. Addressing this question requires moving beyond descriptive host‐range assessments toward integrative, population‐level analyses. Such efforts should include expanded strain sampling across diseased crops and seeds, irrigation systems, surrounding vegetation and environmental reservoirs, combined with genomic analyses.

At the molecular scale, more detailed functional studies of effector repertoires and secretion dynamics are essential. Current evidence indicates that virulence differences among strains cannot be explained solely by the presence or absence of effectors. Instead, coordinated regulation and quantitative tuning of secretion probably play important roles. Integrating comparative genomics, secretome profiling, in planta transcriptomics and targeted mutagenesis will be necessary to distinguish core virulence determinants from accessory traits whose contributions depend on host and environmental context.

Refining diagnostic and surveillance tools is also a priority. Sensitive, lineage‐specific molecular markers combined with epidemiological monitoring could enable earlier differentiation of bacterial leaf spots from visually similar fungal diseases and shift management from reactive intervention to anticipatory and targeted control.

Ultimately, clarifying where *P. syringae* pv. *aptata* falls along the continuum between ecological opportunism and host‐associated adaptation will require integrating phylogenomics, secretion system studies, phyllosphere ecology and field epidemiology into a unified framework. Such efforts may reveal that host association in this lineage is not a static taxonomic trait, but a dynamic ecological outcome shaped by environmental context, regulatory coordination and selective pressures within agroecosystems. In this sense, *P. syringae* pv. *aptata* may serve as a model for understanding how environmentally persistent bacteria become pathogenic in response to shifting ecological and host‐derived signals.

## Author Contributions

**Ivan Nikolić:** conceptualization, writing – original draft, visualization, supervision, project administration. **Iva Rosić:** visualization, writing – review and editing. **Tanja Berić:** writing – review and editing. **Jelena Lozo:** writing – review and editing. **Slaviša Stanković:** writing – review and editing, resources, project administration. **Tamara Ranković:** writing – review and editing. **Marina Sokić:** writing – review and editing, visualization. **Olja Medić:** writing – review and editing.

## Funding

This work was supported by the Ministry of Science, Technological Development and Innovations of the Republic of Serbia, Grants 451‐03‐34/2026‐03/200178 and 451‐03‐33/2026‐03/200178.

## Conflicts of Interest

The authors declare no conflicts of interest.

## Data Availability

The authors have nothing to report.
